# A Possible Mechanism: Genistein Improves Metabolism and Induces White Fat Browning Through Modulating Hypothalamic Expression of *Ucn3, Depp*, and *Stc1*

**DOI:** 10.3389/fendo.2019.00478

**Published:** 2019-07-16

**Authors:** Liyuan Zhou, Xinhua Xiao, Qian Zhang, Jia Zheng, Ming Li, Mingqun Deng

**Affiliations:** Key Laboratory of Endocrinology, Department of Endocrinology, Translational Medicine Center, Ministry of Health, Peking Union Medical College, Peking Union Medical College Hospital, Chinese Academy of Medical Sciences, Beijing, China

**Keywords:** metabolism, white fat browning, hypothalamus, whole transcriptome sequencing, *Ucn3*, *Depp*, *Stc1*

## Abstract

Bioactive food components have gained growing attention in recent years. Multiple studies demonstrated that genistein had beneficial effects on metabolism. However, the exact mechanism by which genistein improves metabolism remains unclear, especially the central regulation. This study was designed to evaluate whether addition of genistein to the high-fat diet could counter metabolic disorders and whether these alterations were associated with gene expression in hypothalamus. C57BL/6 mice were fed either a high-fat diet (HF), high-fat diet with genistein (0.25 g/kg diet) (HFG) or a normal control diet (CON) for 8 weeks. Body weight was assessed during the study. After 8-week intervention, content of inguinal subcutaneous adipose tissue (SAT), perirenal visceral adipose tissue (VAT) and brown adipose tissue (BAT) were weighed. Glucose tolerance test, the serum levels of insulin and lipid were assessed. The mRNA of browning marker was detected in the white fat. The hypothalamus was collected for whole transcriptome sequencing and reverse transcription quantitative PCR validation. The results demonstrated that mice fed HFG diet had lower body weight and SAT mass, decrease levels of low-density lipoprotein cholesterol and free fatty acids, higher browning marker of *Ucp1* and *Cidea* in WAT and an improvement in glucose tolerance and insulin sensitivity compared with those in HF group. Transcriptome sequencing showed that there were three differentially expressed genes in hypothalamus among the three groups, including *Ucn3, Depp*, and *Stc1*, which were significantly correlated with the browning markers in WAT and insulin sensitivity. Thus, regulating gene expressions in hypothalamus is a potential mechanism for genistein improving metabolism and inducing WAT browning, which may provide a novel target for the precaution and treatment of T2DM.

## Introduction

Obesity and type 2 diabetes mellitus (T2DM) have become epidemic worldwide, which desires urgently more effective and novel means to inhibit and slow the occurrence and development of them. In recent years, the health benefits of bioactive food components (1–3), including isoflavones (4, 5), have drawn increasing attention. Soy isoflavone is extensively distributed in leguminous plant and is a plant derived phytoestrogen compound due to similar chemical structure with estrogen. The biological effects of soy isoflavone on the prevention and treatment of cancer (6–9) and other chronic diseases have been widely researched. Epidemiological evidence has shown that the consumption of soy foods and isoflavones is negatively correlated with the risk of T2DM, especially in the obese population (10, 11). Subsequent human (12) and animal studies (13, 14) indicated that dietary supplement with soy protein and isoflavones could significantly improve glucose intolerance, insulin resistance and lipid disorders.

It has been verified that the health benefits of soy isoflavoid are associated with one of its major components, genistein (15). Genistein is a flavonoid mainly derived from soybeans and is also known as a phytoestrogen because of its similar structure to 17β-estradiol. The biological beneficial effects of genistein on cancer (16), cardiovascular disease (17), metabolic syndrome (18, 19), and osteoporosis (20, 21) have been widely investigated in human and animal studies. In the meantime, the safety of genistein intake has been indicated in mice, rats, monkeys and humans (22). Ranging from estrogen-like effects, anti-estrogen effects, protein tyrosine kinase (PTK) inhibitors to antioxidant property, genistein has been proven to have multitude of mechanisms to exert its effects on diseases (22). Although the potential effects of genistein on obesity and glycolipid metabolic disorders have been reported previously in several studies (23, 24), the exact mechanism deciphering the protective effects is still not clearly understood. There is evidence that genistein could directly preserve β-cell function, enhance β-cell proliferation and inhibit apoptosis (22). Furthermore, modulating hepatic glucose and lipid metabolism, regulating cAMP/PKA signaling pathway and epigenetic modification (22) and modifying gut microbiota (25) have all been shown to play potential roles in the effects of genistein on diabetes and dyslipidemia. However, the mechanism investigation of genistein on metabolism was only limited to peripheral tissues, the central regulating mechanism should be paid more attention in light of its crucial role in controlling metabolic health.

In the last few decades, central regulation of metabolic homeostasis gained a growing number of attention in academia. Hypothalamus is the center of regulating food intake and energy homeostasis. Studies have shown that central regulation disorders might play a key role in the development of obesity and metabolic diseases (26, 27). With the rapid development of nutrigenomics, the interaction between nutrients and gene expression is becoming increasingly recognized. Hypothalamic gene expression altered in mice fed a high-fat diet compared with mice fed a normal control diet, including Toll-like receptor 4 (TLR4) associated genes and genes in the IKKβ/NFκB and JNK inflammatory pathways (28). In addition, the regulation of gene expression in hypothalamus by bovine lactoferrin (29), omega-3 fatty acids (30) and proanthocyanidins (31) have been explored in mice fed a high-fat diet. Although there were several studies detecting the changes of hormone levels in hypothalamus after genistein intervention (32, 33), the effects of genistein on hypothalamic gene expression and its role in the metabolic improvement have not been investigated.

Thus, in this study, we aimed to investigate whether genistein could counter the harmful effects of high-fat diets on glucose and lipid metabolism and further explore whether genistein improves metabolic health through modulating the hypothalamic gene expression in mice.

## Materials and Methods

### Animals and Experimental Protocol

Four-week old C57BL/6J female mice purchased from the National Institutes for Food and Drug Control (Beijing, China; SCXK-2014-0013) were housed in the SPF conditions and maintained at 20-24°C and 12 h light/night cycles with access to water and normal control diet (AIN-93G, Research diets, US) ad libitum. After 1 week's acclimation, the mice (*n* = 8 per group) were randomly assigned to three groups and were fed either a high-fat diet (HF), high-fat diet with genistein (CAS: 466-72-0, G0272, TCI Development Co., Ltd.) (0.25 g/kg diet) (HFG) or normal control diet (CON). The nutrient composition was shown in Table S1. The HF diet included 60% of calories from fat, whereas the CON diet contained 15.8% of calories derived from fat. And the calorific value of HF diet and CON diet are 5.24 and 3.9 kcal/g, respectively. After 8 weeks of genistein treatment, the blood samples were collected from the intraorbital retrobulbar plexus after 10-h of fasting. The subcutaneous adipose tissue (inguinal adipose tissue) (SAT), visceral adipose tissue (perirenal adipose tissue) (VAT) and brown adipose tissue (interscapular adipose tissue) were removed and weighed after mice were euthanized. The hypothalamus was removed as previously described (34) and then stored at −80°C for further analysis. During the experiment, body weight and food intake were assessed once a week. All operations were conducted under anesthesia, and best efforts were done to minimize suffering. All animal protocols were approved by the institutional animal care and use committee of the Peking Union Medical College Hospital (Beijing, China, SYXK-2018-0019). All of the animal operations were performed in compliance with the Guide for the Care and Use of Laboratory Animals.

### Glucose Tolerance Test (GTT)

At the end of treatment, intraperitoneal glucose tolerance test (IPGTT) was performed. After fasted for 6 h, the mice were injected intraperitoneally with a glucose load of 2 g/kg body weight. The blood glucose levels were measured in tail vein before (0 min), 30, 60, and 120 min after the injection using a Contour TS glucometer (ACCU-CHEK Mobile, Beijing, China). In addition, the area under the curve (AUC) of the IPGTT was calculated (35).

### Measurement of Biochemical Parameters

Blood samples were collected after 8 weeks' intervention and were centrifuged at 3,000 × g for 10 min at 4°C. The serum total cholesterol (TC), triglycerides (TG), low-density lipoprotein cholesterol (LDL-C), high-density lipoprotein cholesterol (HDL-C), and free fatty acids (FFA) were measured by routine automated laboratory methods. The fasting insulin levels were detected using a mouse insulin ELISA kit (80-INSMSU-E01, Salem, NH, USA). The homeostasis model assessment of insulin resistance (HOMA-IR) was used to assess the insulin sensitivity. And the calculation of HOMA-IR was the same as previously described (35).

### RNA Preparation and Whole Transcriptome Sequencing

The gene expression levels in the hypothalamus tissues were detected using whole transcriptome sequencing analyses (*n* = 3 per group). TRIzol reagent (Life Technologies Inc., Carlsbad, CA, USA) was used to extract total RNA from the hypothalamus tissues. The degradation and contamination of RNA was monitored on 1% agarose gels. RNA concentration was measured using Qubit RNA Assay Kit in Qubit 2.0 Flurometer (Life Technologies, CA, USA). All RNA samples had high quality without degradation and contamination. Two microgram RNA per sample was used as input material for the RNA library preparations. NEBNext UltraTM RNA Library Prep Kit for Illumina (NEB, USA) was used to generate sequencing libraries and index codes were added to attribute sequences to each sample. The clustering of the index-coded samples was performed on a cBot Cluster Generation System using TruSeq PE Cluster Kit v4-cBot-HS (Illumia) according to the manufacturer's instructions. After cluster generation, the library preparations were sequenced on an Illumina Hiseq 4,000 platform and paired-end 150 bp reads were generated. After quality control, the clean reads were mapped to the reference genome sequence (Mus musculus (assembly GRCm38.p6), NCBI) using Tophat2 tools soft. Differential expression analysis of two groups was performed using the DESeq R package (1.10.1). *P*-value < 0.05 found by DESeq were considered as differentially expressed. To discover the biological significance of the altered genes, the enrichment of differentially expressed genes in KEGG (Kyoto Encyclopedia of Genes and Genomes) pathways was analyzed using KOBASsoftware.

### Reverse Transcription Quantitative PCR (RT-qPCR) Experiment

To validate the results of whole-transcriptome sequencing, we selected the three differentially expressed genes for RT-qPCR analysis (*n* = 6 per group). In addition, in order to determine whether genistein could improve the metabolism of adipose tissue and brown the white fat, we detect five genes associated with browning in inguinal adipose tissue, including *Ucp1* (uncoupling protein 1), *Cidea* (cell death activator), *PGC1*α (peroxisome proliferator-activated receptor gamma coactivator 1-alpha), *PPAR*α (peroxisome proliferator-activated receptor alpha), and *PPAR*γ (peroxisome proliferator-activated receptor gamma) (*n* = 6 per group).

Total RNA was prepared as mentioned above. Then, 1.0 μg of total RNA was reverse transcribed into cDNA using the PrimeScript™ RT reagent Kit with gDNA Eraser (RR047A, TaKaRa Bio Inc., Otsu, Shiga, Japan). cDNA (2 μl) was amplified on an ABI 7500 thermocycler (Applied Biosystems, CA, USA) using the TB Green PCR Master Mix (RR820A, Takara Bio Inc., Otsu, Shiga, Japan) in a total reaction volume of 20 μL. The reaction conditions included initial denaturation step (30 s at 95°C) and cycling step (denaturation for 5 s at 95°C and annealing and extending for 34 s at 60°C for 40 cycles). β-actin was used for normalization. All the sequences of the primers were listed in Table S2. The relative expression levels of the genes were quantified by 2^−ΔΔ*Ct*^ method.

### Statistical Analysis

All analyses were performed using Prism version 7.0 (GraphPad Software Inc., San Diego, CA, USA). Data are presented as the means ± standard error of the means (S.E.M). Multiple comparisons between groups were analyzed using one-way ANOVA and two-way ANOVA, with Tukey and Bonferroni *post hoc* analyses. The relationships between gene expression in hypothalamus and browning markers as well as insulin sensitivity were analyzed by spearman correlation analysis. A *p-*value < 0.05 was considered as statistical significance.

## Results

### Body Weight

**Figure 1:**
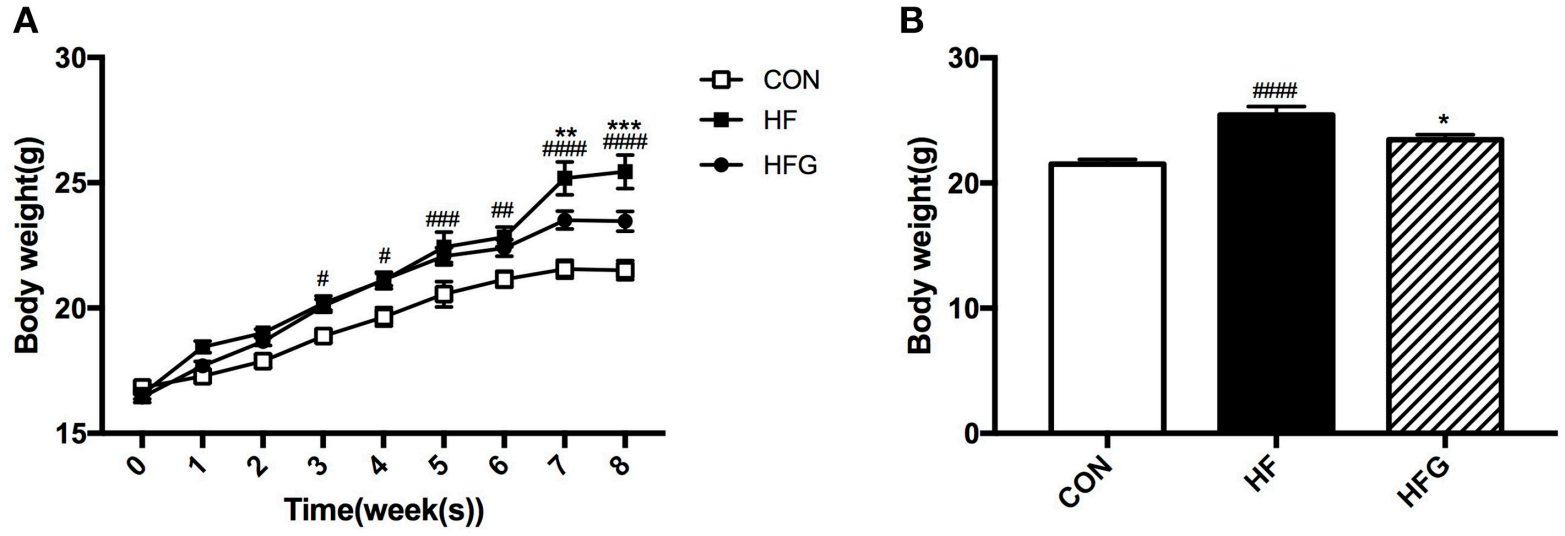
The alterations of body weight among the three groups during the 8-week of genistein intervention. **(A)** the changes of body weight; **(B)** body weight by the end of the study. CON, normal control diet; HF, high-fat diet; HFG, high-fat diet with genistein. Data are expressed as means ± S.E.M. (*n* = 8/group). Mean values were significantly different between the HF group and the CON group: ^#^*p* < 0.05; ^##^*p* < 0.01; ^###^*p* < 0.001; ^####^*p* < 0.0001. Mean values were significantly different between HF group and the HFG group: *p < 0.05, **p < 0.01, ***p < 0.001.

First, we detected the effects of three different dietary interventions on the body weight. Figure 1A shows the changes of body weight during the 8 weeks among the three groups. The body weight in mice of the HF group has been higher than that of the CON group since the third week of intervention (*p* < 0.05). Interestingly, genistein feeding could significantly reduce body weight induced by a HF diet until the seventh week of treatment (*p* < 0.01). At the end of the intervention, the body weight in mice of the HFG group was dramatically decreased compared with that of the HF group (*p* < 0.05) (Figure 1B). Although the marked differences in the body weight, there were no differences in the food intake among the three groups (data not shown).

### Lipid Metabolism

In order to evaluate whether significant reduction in body weight of mice fed a HFG diet compared to that fed a HF diet affected the body composition of mice, we weighed the adipose tissue of mice among the three groups (Table 1). The absolute and relative content of SAT (*p* < 0.01; *p* < 0.0001) and VAT (*p* < 0.01; *p* < 0.01) were both significantly higher in mice fed a HF diet than mice fed control diet. Genistein led to a decrease in the content of SAT (*p* < 0.05; *p* < 0.01) and VAT (*p* > 0.05). However, no significant differences were identified among the three groups in terms of the content of BAT. Furthermore, we explored the effects of genistein on the serum lipid profiles. The levels of serum TC (*p* < 0.01) and LDL-C (*p* < 0.01) were significantly higher in mice of HF group than that of CON group. Genistein markedly reduced the serum levels of LDL-C (*p* < 0.01) and FFA (*p* < 0.05).

**Table 1:** Parameters of lipid metabolism.

**Parameters**	**CON (*n* = 8)**	**HF (*n* = 8)**	**HFG (*n* = 8)**
SAT (g)	0.091 ± 0.010	0.294 ± 0.060**^##^**	0.158 ± 0.022^*^
SAT (%)	0.428 ± 0.048	1.243 ± 0.157**^####^**	0.667 ± 0.0873^**^
VAT (g)	0.040 ± 0.009	0.161 ± 0.041**^##^**	0.088 ± 0.013
VAT (%)	0.185 ± 0.040	0.644 ± 0.141**^##^**	0.400 ± 0.032
BAT (g)	0.064 ± 0.009	0.118 ± 0.021	0.081 ± 0.008
BAT (%)	0.361 ± 0.055	0.405 ± 0.043	0.343 ± 0.030
TC (mmol/l)	2.425 ± 0.189	3.188 ± 0.077**^##^**	2.645 ± 0.179
TG (mmol/l)	0.205 ± 0.038	0.220 ± 0.036	0.131 ± 0.028
LDL-C (mmol/l)	0.234 ± 0.024	0.330 ± 0.015**^##^**	0.223 ± 0.014^**^
HDL-C (mmol/l)	1.270 ± 0.045	1.313 ± 0.030	1.357 ± 0.028
FFA (umol/l)	1534.000 ± 85.570	1653.000 ± 112.600	1313.000 ± 78.610^*^

*SAT, subcutaneous adipose tissue; VAT, visceral adipose tissue; BAT, brown adipose tissue; TC, total cholesterol; TG, triglyceride; HDL-C, high-density lipoprotein cholesterol; LDL-C, low-density lipoprotein cholesterol; FFA, free fatty acids; CON, normal control diet; HF, high-fat diet; HFG, high-fat diet with genistein. Data are expressed as means ± S.E.M. (n = 8/group). Mean values were significantly different between the HF group and the CON group*:

## *p < 0.01*;

#### *p < 0.0001. Mean values were significantly different between the HF group and the HFG group*:

* *p < 0.05*,

** *p < 0.01*.

### Browning of the Subcutaneous White Adipose Tissue

**Figure 2:**
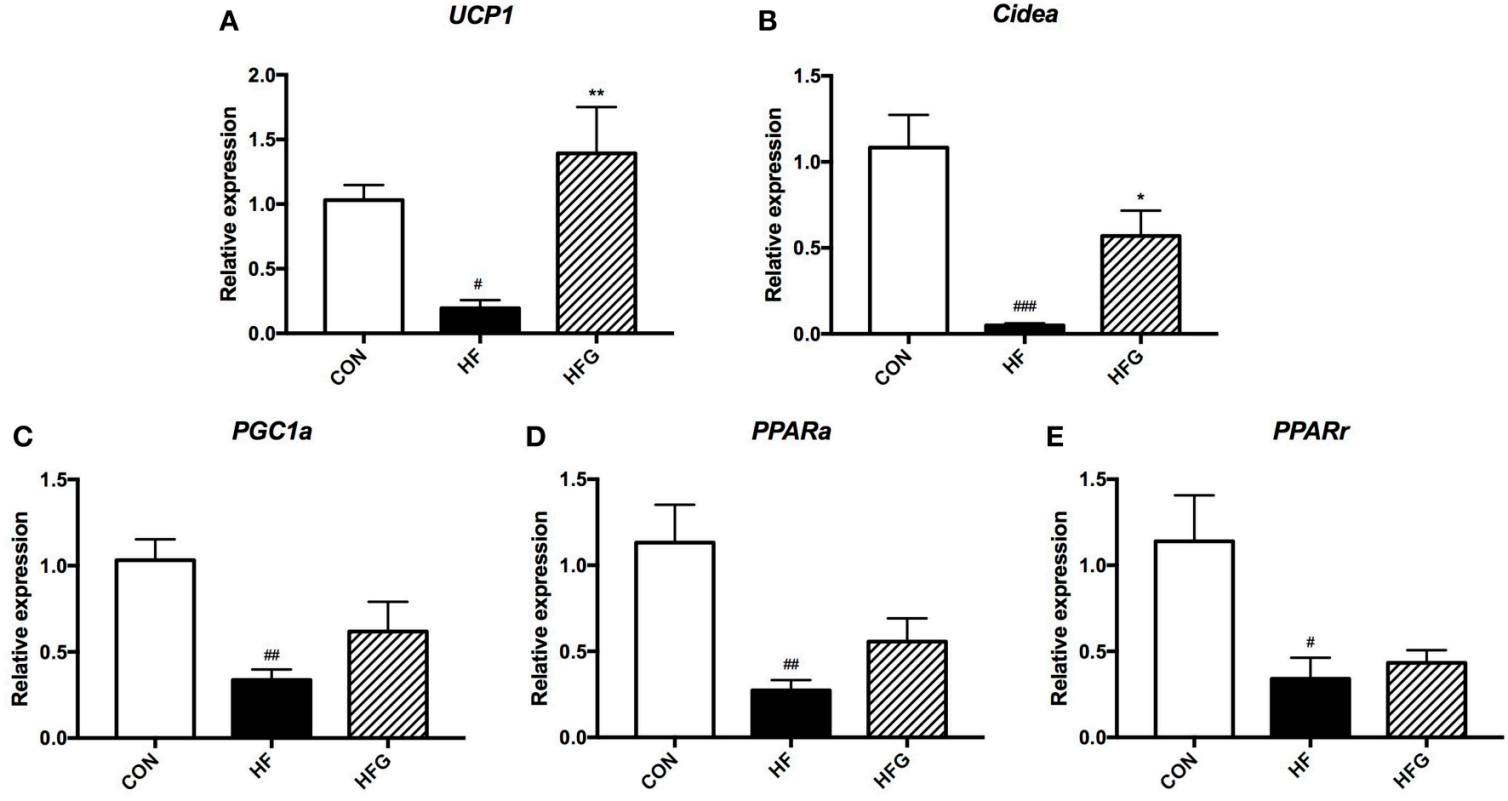
The relative gene expression levels of browning marker in the inguinal adipose tissue. **(A)** *Ucp1*; **(B)** *Cidea*; **(C)** *PGC1*α; **(D)** *PPAR*α*;* and **(E)** *PPAR*γ. CON, normal control diet; HF, high-fat diet; HFG, high-fat diet with genistein. Data are expressed as means ± S.E.M. (*n* = 6/group). Mean values were significantly different between the HF group and the CON group: ^#^*p* < 0.05; ^##^*p* < 0.01; ^###^*p* < 0.001. Mean values were significantly different between HF group and the HFG group: *p < 0.05, **p < 0.01.

Although there were no effects of genistein on the content of BAT, we next assessed whether genistein intervention could activate subcutaneous white adipose tissue browning in light of its evident decreases in SAT mass and significant improvement in lipid metabolism. As shown in Figure 2, the mRNA expression of browning markers was significantly decreased in mice fed a HF diet compared to that fed a control diet, including *Ucp1* (*p* < 0.05), *Cidea* (*p* < 0.001), *PGC1*α (*p* < 0.01), *PPAR*α (*p* < 0.01), and *PPAR*γ (*p* < 0.05). Interestingly, the HFG diet could dramatically increase the expression of *Ucp1* (*p* < 0.01) and *Cidea* (*p* < 0.05) in subcutaneous WAT (Figures 2A,B). Thus, the reduction in adipose tissue mass by genistein was associated with the increased browning of WAT.

### Glucose Metabolism and Insulin Sensitivity

**Figure 3:**
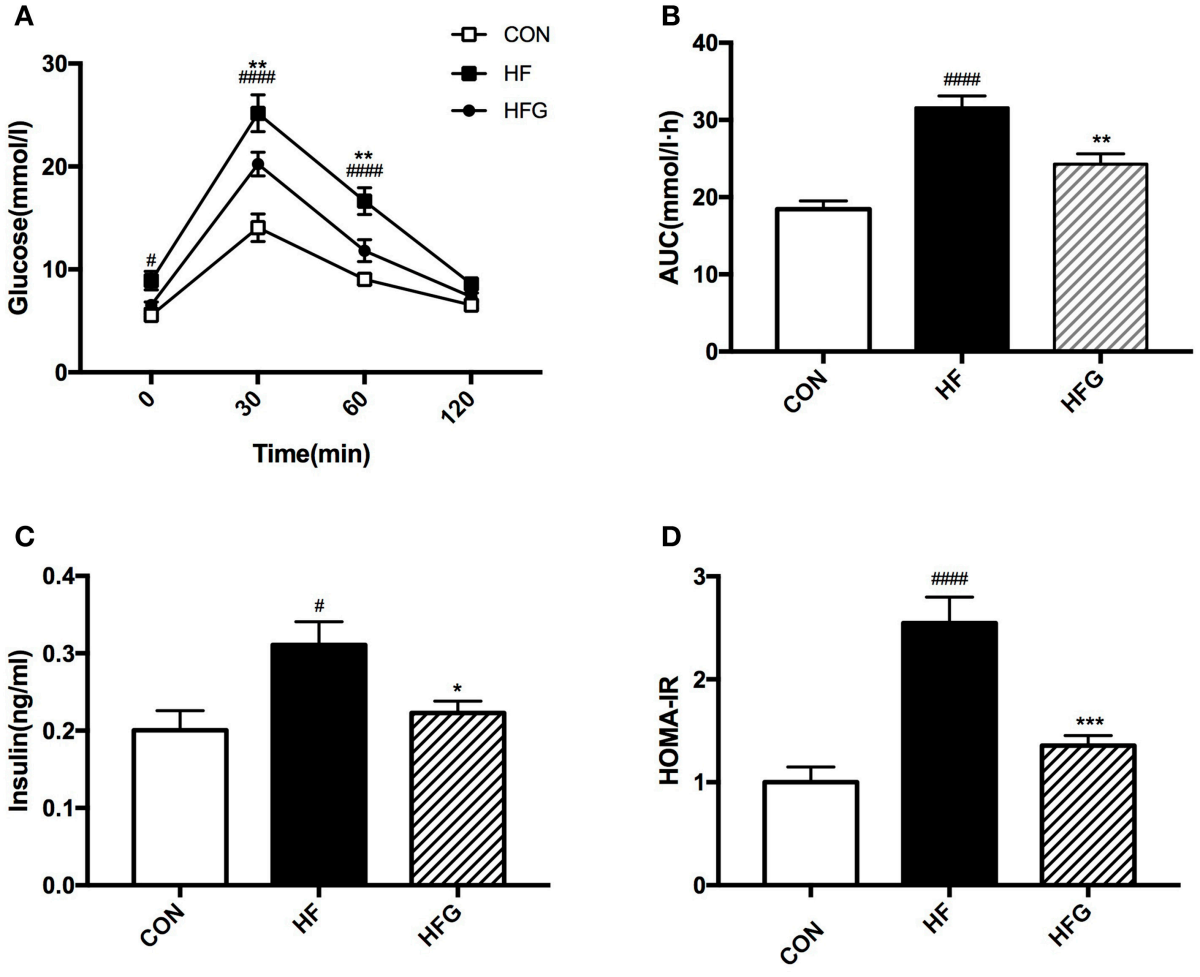
Glucose tolerance and insulin sensitivity of mice. **(A)** IPGTT; **(B)** AUC; **(C)** serum insulin levels; **(D)** HOMA-IR. IPGTT, intraperitoneal glucose tolerance test; AUC, area under the curve; HOMA-IR, the homeostasis model assessment of insulin resistance. CON, normal control diet; HF, high-fat diet; HFG, high-fat diet with genistein. Data are expressed as means ± S.E.M. (*n* = 8/group). Mean values were significantly different between the HF group and the CON group: ^#^*p* < 0.05; ^####^*p* < 0.0001. Mean values were significantly different between HF group and the HFG group: *p < 0.05, **p < 0.01, ***p < 0.001.

We then evaluated whether decreases in adipose tissue mass and increases in WAT browning could modulate glucose homeostasis and insulin sensitivity. At the end of treatment, the HF diet fed mice had significantly impaired glucose tolerance compared with mice in the CON group, which is characterized by higher blood glucose levels at 0 min (*p* < 0.05), 30 min (*p* < 0.0001), and 60 min (*p* < 0.0001) during the IPGTT (Figure 3A) and significantly lager AUC of IPGTT (*p* < 0.0001) (Figure 3B). In order to determine whether genistein intake could fight against the deleterious effects of HF on the glucose tolerance, we compared the blood glucose levels of mice in the HFG group and the HF group. The results showed that the blood glucose levels at 30 min (*p* < 0.01) and 60 min (*p* < 0.01), and AUC (*p* < 0.01) of mice fed genistein were dramatically lower (Figures 3A,B). In addition to glucose tolerance, insulin sensitivity of mice was also evaluated. As shown in Figures 3C,D, HF diet resulted in a markedly higher fasting insulin levels (*p* < 0.05) and larger HOMA-IR index (*p* < 0.0001) in mice than mice fed a control diet. However, genistein fed mice showed an evident improvement in insulin sensitivity. Significantly decreased insulin levels (*p* < 0.05) and smaller HOMA-IR index (*p* < 0.001) were observed in the HFG group compared with that in the HF group.

### Gene Expression in the Hypothalamus

**Figure 4:**
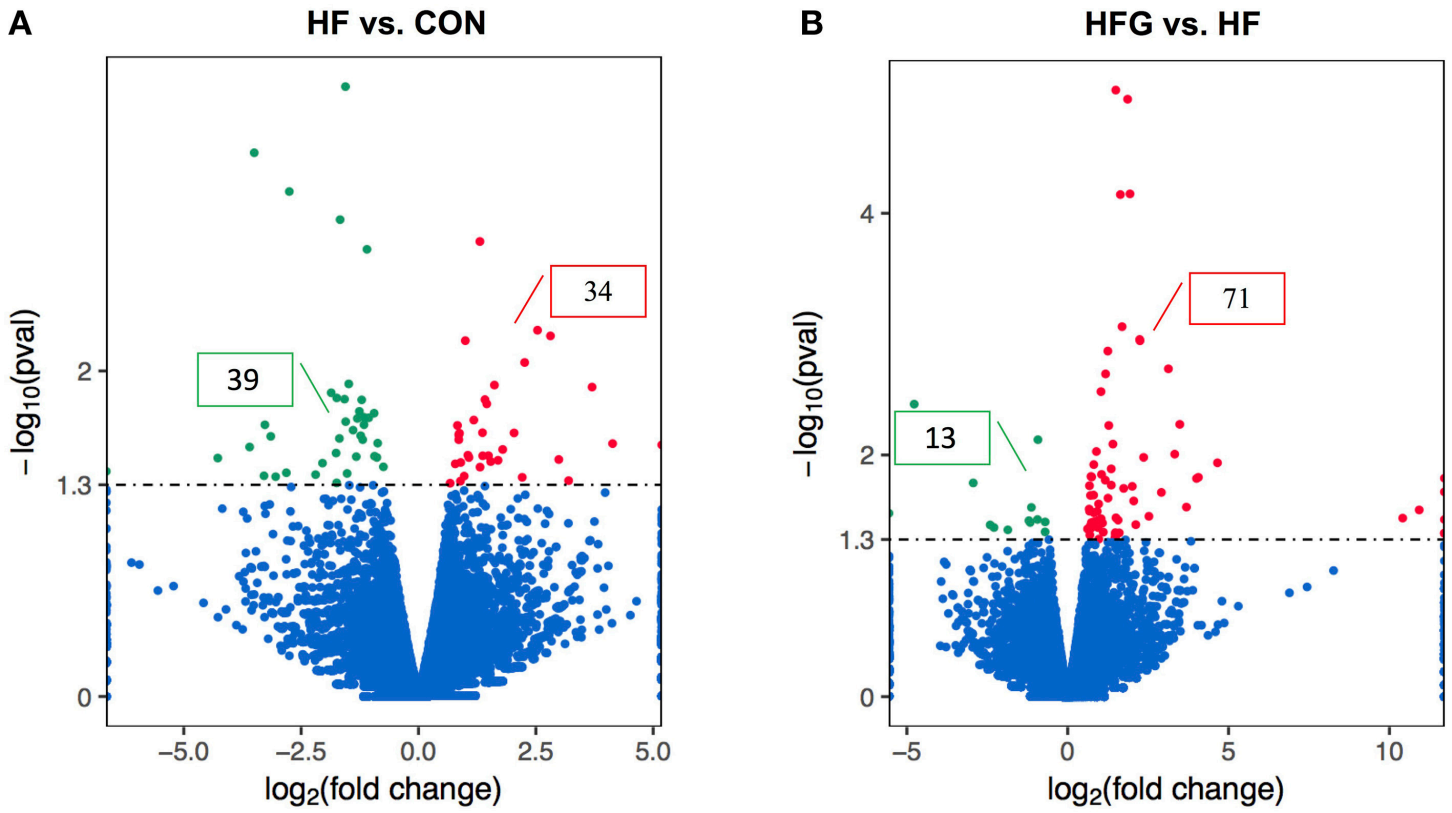
The differentially expressed genes in the hypothalamus. The Volcano Plot graphs show the log2 of the fold change in each gene's expression between two groups and its -log (*p*-value) from the *t*-test. The red indicates that the gene is up-regulation, whereas the green indicates that gene is down-regulation. **(A)** HF vs. CON; **(B)** HFG vs. HF. CON, normal control diet; HF, high-fat diet; HFG, high-fat diet with genistein (*n* = 3/group).

**Figure 5:**
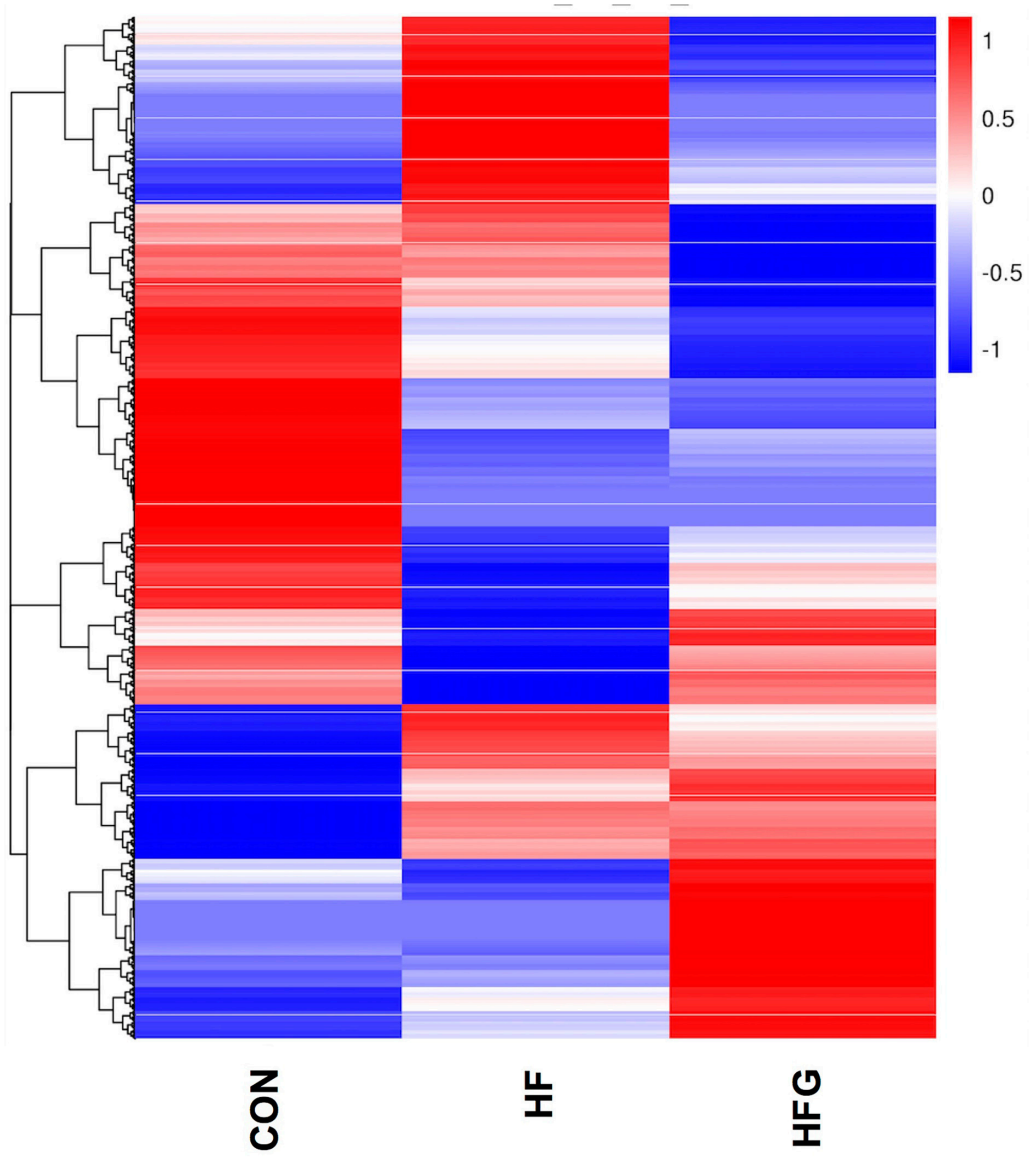
The heatmap diagram of the differentially expressed genes in hypothalamus among the three groups. CON, normal control diet; HF, high-fat diet; HFG, high-fat diet with genistein (*n* = 3/group).

In light of the central role of hypothalamus in maintaining the homeostasis of food intake and energy expenditure, we assessed whether genistein intake improved metabolism through regulating hypothalamic gene expression. The sequence data has been submitted to the Sequence Read Archive (SRA) database (accession number SRP187329). The results of whole transcriptome sequencing showed that there were 73 differentially expressed genes identified in the hypothalamic tissues between the HF group and the CON group, among which 39 genes were down-regulated, while the other 34 genes were up-regulated in the HF group (Figure 4A). While in comparison to the gene expression in mice fed a HF diet, 84 genes were differentially expressed in hypothalamus of mice fed a HFG diet, including 13 down-regulated genes as well as 71 up-regulated genes (Figure 4B). Figure 5 showed the hierarchical clustering of the differentially expressed genes in hypothalamus of mice fed the three different dietary intervention. Then, to identify the possible pathways modulated by genistein in hypothalamus, we analyzed the KEGG pathways enriched by the differentially expressed genes between the HF group and the HFG group. As shown in Table 2, the “Phosphatidylinositol signaling system” (*p* = 0.0029), “Neuroactive ligand-receptor interaction” (*p* = 0.0090), “Glycolysis/Gluconeogenesis” (*p* = 0.0158), “Inositol phosphate metabolism” (*p* = 0.0176), “Metabolic pathways” (*p* = 0.0303), and some other pathways were significantly enriched.

**Table 2:** Kyoto Encyclopedia of Genes and Genomes (KEGG) pathways of differentially expressed genes between HF group and the HFG group (*p* < 0.05).

**KEGG ID**	**Pathway Name**	**O**	**C**	***p*-value**
04070	Phosphatidylinositol signaling system	3	97	0.0029
04080	Neuroactive ligand-receptor interaction	4	285	0.0090
00010	Glycolysis/gluconeogenesis	2	66	0.0158
00562	Inositol phosphate metabolism	2	70	0.0176
04350	TGF-beta signaling pathway	2	85	0.0251
01100	Metabolic pathways	8	1,298	0.0303

*O, the number of differential expression genes in the pathway; C, the number of reference genes in the pathway*.

**Figure 6:**
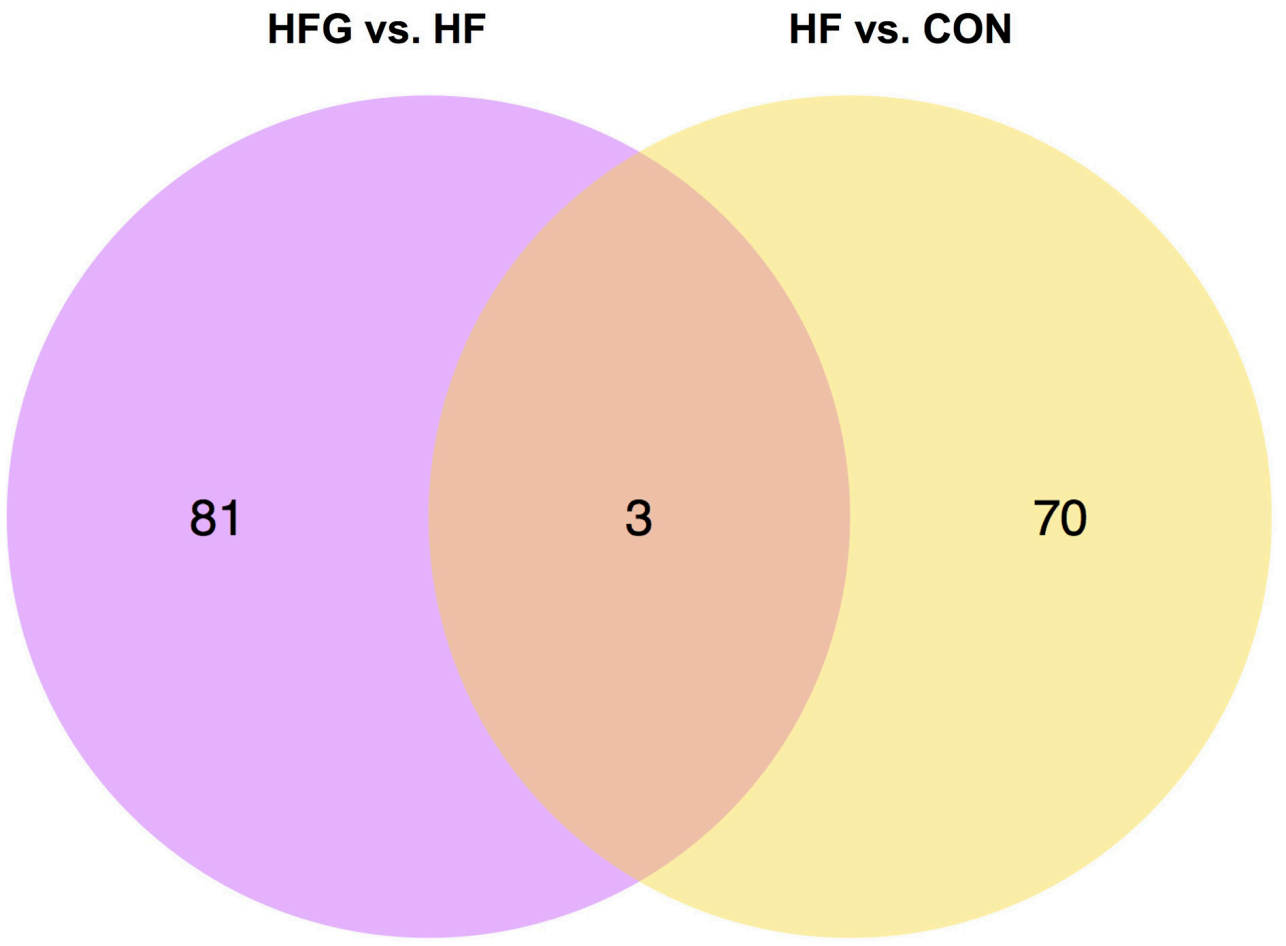
The Venn diagram of the differentially expressed genes in hypothalamus. CON, normal control diet; HF, high-fat diet; HFG, high-fat diet with genistein (*n* = 3 per group).

To explore the specific mechanism that genistein improves metabolism and activates WAT browning, we further analyzed the differentially expressed genes among the three groups. Figure 6 showed that there were three common differentially expressed genes between HF vs. CON group and HFG vs. HF group, including *Ucn3* (urocortin 3), *Depp* (decidual protein induced by progesterone), and *Stc1* (stanniocalcin1). They were all significantly lower expression in mice fed a HF diet than that fed a control diet. In contrast, HFG diet markedly up-regulated the expression of all the three genes in hypothalamus of mice compared with that of mice in the HF group. These results indicated that regulating the hypothalamic gene expression might be one of the mechanism that genistein improved metabolism in mice fed a HF diet.

### RT-qPCR Validation of the Whole Transcriptome Sequences of the Hypothalamus

**Figure 7:**
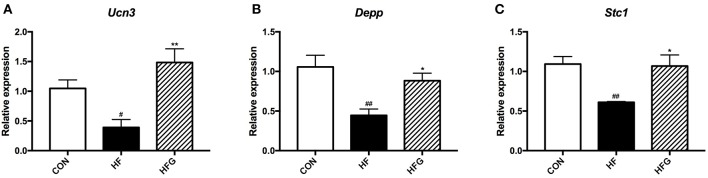
RT-qPCR verification of differentialy expressed genes among the three groups. **(A)** *Ucn3*; **(B)** *Depp*; and **(C)** *Stc1*. CON, normal control diet; HF, high-fat diet; HFG, high-fat diet with genistein. Data are expressed as means ± S.E.M. (*n* = 6/group). Mean values were significantly different between the HF group and the CON group: ^#^*p* < 0.05; ^##^*p* < 0.01. Mean values were significantly different between HF group and the HFG group: *p < 0.05, **p < 0.01.

In order to verify the sequencing results, we selected the three differentially expressed genes, including *Ucn3, Depp*, and *Stc1* for verification using RT-qPCR. As shown in Figure 7, the *Ucn3* (*p* < 0.05), *Depp* (*p* < 0.01), and *Stc1* (*p* < 0.01) were significantly down-regulated in the HF group compared with the CON group. However, the intake of genistein could dramatically increased the expression of all the three genes, which indicated that these genes played crucial roles in the beneficial effects of genistein on metabolism. Thus, a marked consistency was observed between the transtriptome sequencing and the RT-qPCR results, which validated the reliability of our whole transtriptome sequences.

### Correlation Analysis Between the Differentially Expressed Genes in Hypothalamus and Browning Markers in WAT as Well as Insulin Sensitivity

**Figure 8:**
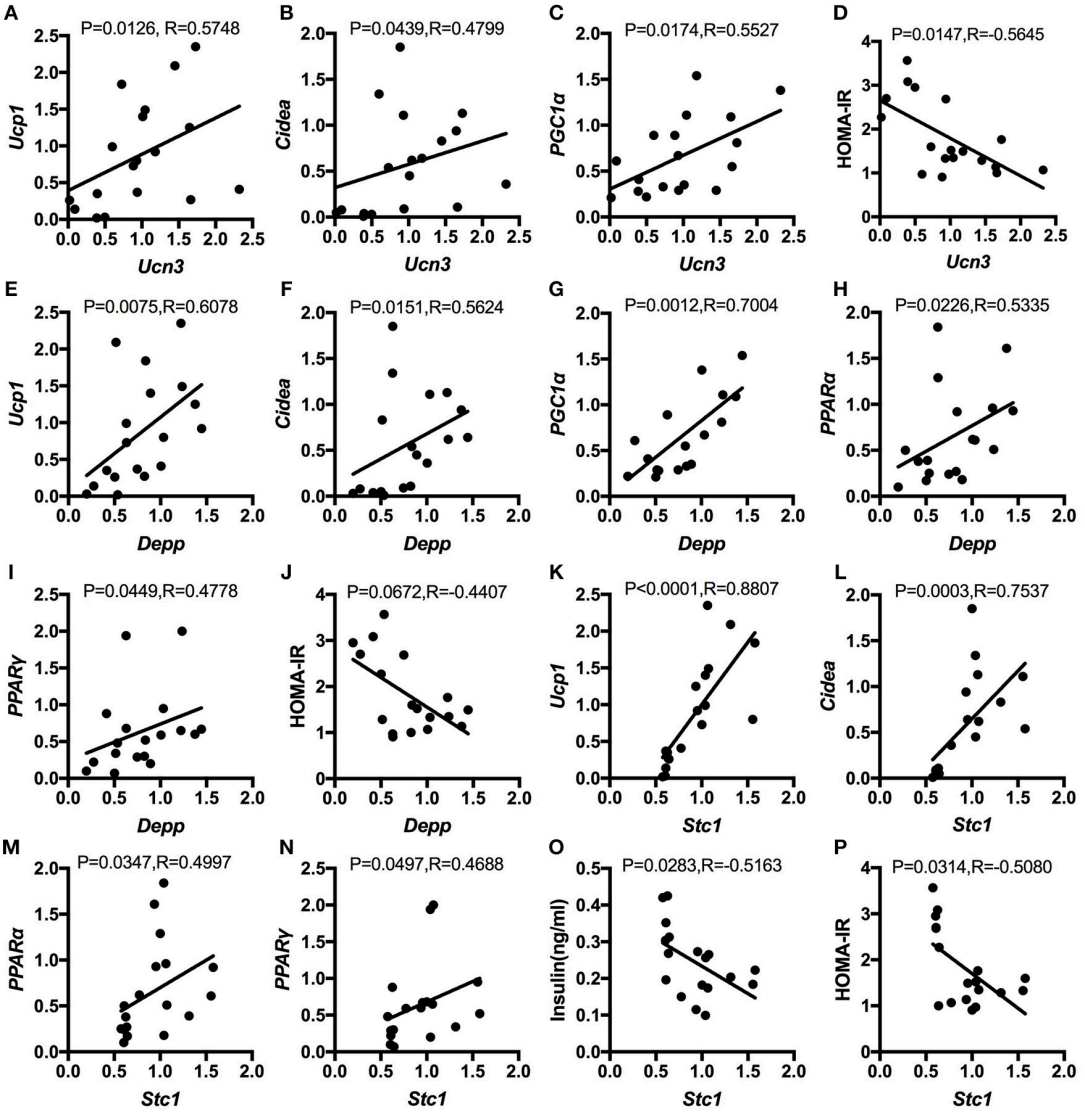
Spearman correlation analysis between the relative expression of the altered genes in hypothalamus and the relative expression of browning markers in WAT as well as insulin sensitivity. **(A)**; *Ucn3* and *UCP1*; **(B)** *Ucn3* and *Cidea*; **(C)** *Ucn3* and *PGC1*α; **(D)** *Ucn3* and HOMA-IR; **(E)** *Depp* and *UCP1*; **(F)** *Depp* and *Cidea*; **(G)** *Depp* and *PGC1*α; **(H)** *Depp* and *PPAR*α; **(I)** *Depp* and *PPAR*γ; **(J)** *Depp* and HOMA-IR; **(K)** *Stc1* and *UCP1*; **(L)** *Stc1* and *Cidea*; **(M)** *Stc1* and *PPAR*α; **(N)** *Stc1* and *PPAR*; **(O)** *Stc1* and serum insulin levels; and **(P)** *Stc1* and HOMA-IR.

Next, to explore the relationship between hypothalamic gene expression and WAT browning as well as insulin sensitivity, we did the spearman correlation analysis. The results showed that up-regulated *Ucn3, Depp*, and *Stc1* by genistein intake were all positively correlated with the relative expression of browning markers, including the *UCP1, Cidea, PGC1*α, *PPAR*α, and *PPAR*γ. In addition, the elevated expression of *Ucn3, Depp*, and *Stc1* in hypothalamus of HFG fed mice were negatively related with the serum insulin levels or the HOMA-IR index (Figure 8).

## Discussion

The development of obesity and T2DM are unstoppable throughout the world. The beneficial effects of bioactive compounds on metabolism have gained growing attention. Genistein, one of the most abundant components in soy-derived isoflavones that shares similar structure with estrogen, has been shown to have multitude of biological effects. In the present study, the results showed that addition of isoflavoid genistein to the HF diet could significantly reduce the body weight and the subcutaneous adipose tissue mass. In addition, these decreases were accompanied by increase browning of the SAT, improved serum lipid disorders, and better glucose tolerance and insulin sensitivity. The benefits of genistein on glucose and lipid metabolism are in agreement with the observations of the previous studies (19, 24, 25).

Subcutaneous adipose tissue is more associated with adipose tissue browning than other fats due to more abundant beige adipocytes in the SAT, especially in the inguinal adipose tissue (36). It is known that the brown adipose tissue is responsible for generating heat to maintain body temperature and consuming excessive energy to keep balance of energy expenditure, which might play crucial roles in fighting against metabolic diseases. Our results also indicated that 8 weeks of genistein consumption (0.25 g/kg diet) could significantly activate browning of the inguinal adipose tissue, which was supported by the increase expression of the brown adipose tissue specific genes-*Ucp1* and *Cidea* (37, 38). This finding is also consistent with a recent study, while Tovar's study fed the mice with higher dose of genistein (2 g/kg diet) for a longer time (6 months). Thus, this study demonstrated that dietary genistein could improve metabolism through increasing energy expenditure in a lower dose.

It is well-known that the hypothalamus serves as a center in regulating homeostasis of food intake and energy expenditure. It is composed of multiple nuclei that integrates and mediates the communication between the brain center and peripheral metabolic organs, especially the adipose tissue. There is evidence that the hypothalamus plays important roles in regulating activity of brown adipose tissue and thermogenesis. The hypothalamic nuclei, including ventromedial hypothalamus (VMH), dorsomedial hypothalamus (DMH), paraventricular nucleus (PVN), lateral hypothalamus (LH), and arcuate nucleus (ARC), were all regarded as the potential region of regulating BAT thermogenesis and browning of WAT (39–41). Therefore, the hypothalamic pathways and the complete neural mechanisms in the control of thermoregulation seems to be complicated and remains to be elucidated. In the present study, we detected the whole transcriptome sequences in the hypothalamus. KEGG pathway analysis showed that the altered genes after genistein intervention significantly enriched in the Glycolysis/Gluconeogenesis, Metabolic pathways, and some other pathways. Thus, dietary genistein intake could modulate metabolic pathways in hypothalamus. More interestingly, we also found three common differentially expressed genes among the three groups, including *Ucn3, Depp*, and *Stc1*. Correlation analysis showed that these three genes were all significantly related with the browning markers in WAT and insulin sensitivity. This may be a novel mechanism to decipher the effects of genistein on WAT browning and metabolism.

There are corresponding nuclei in hypothalamus regulating appetite through producing orexigenic peptides, including agouti-related protein (AGRP) and neuropeptide Y (NPY), and anorexigenic peptides, such as proopiomelanocortin (POMC). Urocortin 3 (UCN3), a 38-amino acid peptide, is a member of corticotropin-releasing hormone (CRH) family of peptides, which was discovered in 2001 (42). *Ucn3* is highly expressed in the brain of medial amygdala and hypothalamus in human and rodents and is the ligand with high affinity to the corticotropin-releasing factor receptor 2 (CRFR2) (43). Central administration of UCN3 were found to significantly inhibit appetite by reducing food intake (44, 45). Thus, UCN3 is referred to as another anorexigenic peptide. In addition, UCN3/CRHR2 possesses a variety of biological effects in the regulation of stress and anxiety (46), as well as stimulating sympathetic outflow, which was characterized by increased thermogenesis of BAT (47) and upregulated *Ucp1* in BAT (48). In addition, another study showed that global *Ucn3* knockout increased food intake and reduced insulin sensitivity (49). Consistent with the previous study, in the present study, we found that the hypothalamic expression of *Ucn3* was inhibited by eight-week of HF diet, while the supplementation of genistein countered the harmful effects of HF diet and up-regulated the *Ucn3*, which was associated with improved glucose and lipid metabolism, insulin sensitivity and browning of inguinal WAT. Therefore, the increased expression of *Ucn3* in hypothalamus might activate WAT browning and increase energy expenditure in peripheral tissues by stimulating sympathetic flow, which might play an important role in mediating the metabolic benefits of genistein consumption.

Decidual protein induced by progesterone (*Depp*) was initially discovered by Watanabe et al. from the human endometrial stromal cells (ESC) cDNA library that is mainly induced by progesterone in ESCs during decidualozation and placental development (50). The significant role of DEPP in the initiation of autography has been indicated (51). In addition, recent studies showed that DEPP was also an insulin-regulatory molecule. The overexpression of *Depp* in insulin-sensitive organs, including liver and white adipose tissue, could reduce the insulin level, inhibit lipogenesis and gluconeogenesis and promote fatty acid oxidation. Elevated reactive oxygen species (ROS) levels inducing fibroblast growth factor 21 (FGF21) in liver might be the potential mechanism (52, 53). It has been found that the *Depp* mRNA is expressed in the liver, adipose tissue, kidney, heart and some other peripheral tissues both in humans and mice. However, the expression of *Depp* in brain is still unknown. In the present study, we firstly found that the *Depp* was also expressed in the hypothalamus of mice. Consistent with the findings in liver and WAT, mice fed a HF diet with a higher level of insulin had lower expression of *Depp* compared with that fed a control diet. After genistein intervention, the insulin resistance was improved which was accompanied by significant increase in the *Depp* expression. And the expression of *Depp* was positively correlated with the browning markers while negatively related with HOMA-IR. Thus, the expression level of *Depp* in the hypothalamus might play an important role in the metabolic benefits of genistein supplementation. However, the exact regulating mechanisms in hypothalamus remain largely unknown and need further investigations.

Stanniocalcin 1 (STC1) is a highly evolutionarily conserved glycoprotein hormone that plays crucial roles in regulating levels of calcium and phosphate in fish (54). However, STC1 is ubiquitously expressed in mammals, including the ovary, skeletal muscle, heart, brain, lung, pancreas, and kidney and has more metabolically associated functions (55). Although it was thought to function in an autocrine or paracrine manner, recent evidence indicated that the mammalian STC1 was also blood-borne (56). Thus, the biological function of STC1 is considered to be intricate and yet to be defined. Previous studies have investigated the role of STC1 in the heart failure (57) and cancer (58). In terms of energy metabolism, *Stc1* transgenic mice showed lower body weight and increased glucose clearance (59). Schein et al. found that STC1 could inhibit renal gluconeogenesis in rat (60). In addition, Zaidi et al. discovered that STC1 co-localized with insulin in mouse islets and was an important aspect of insulin release (61). Different methylation of *Stc1* was one of the genome sites influenced by maternal diabetes during pregnancy, which was associated with impaired insulin secretion and higher risk of T2DM (62). Therefore, STC1 is a potential protein in regulating metabolic health. A large number of studies have confirmed that activation of the mitochondrial antioxidant pathway and inhibition of inflammation are possible protective mechanisms of STC1 on metabolism (63, 64). To our knowledge, this is the first study that investigating the expression of *Stc1* in mice hypothalamus and its role in the metabolic benefits. We found that genistein intake could counter the reduced expression of *Stc1* in hypothalamus induced by the HF diet, which might play important roles in fighting against the inflammation reaction in mice fed a HF diet and further improves metabolism. However, the specific mechanism that hypothalamic STC1 regulates metabolism is still unclear, although our study indicated the association between *Stc1* mRNA expression with browning markers in WAT as well as insulin sensitivity.

In conclusion, our research provides evidence that the consumption of genistein could fight against the deleterious effects induced by the HF diet on glucose and lipid metabolism. Furthermore, increasing energy expenditure by activating browning of SAT was another metabolic benefit in mice fed the genistein. To the best of our knowledge, this is the first study finding that modulating the gene expression of *Ucn3, Depp*, and *Stc1* in hypothalamus might be a crucial mechanism for genistein improving metabolism. However, there were several limitations in this study. The specific mechanism that hypothalamic genes regulate the peripheral metabolism is unclear of yet and needs further exploration in future. Adding inhibitors to the genes or the proteins could help to validate this finding. In addition, we only analyzed the browning of WAT in this study. Detecting the energy expenditure and body temperature of the mice after genistein intake is helpful to understand the benefits of genistein on metabolism.

## Data Availability

The raw data supporting the conclusions of this manuscript will be made available by the authors, without undue reservation, to any qualified researcher.

## Ethics Statement

All animal protocols were approved by the institutional animal care and use committee of the Peking Union Medical College Hospital (Beijing, China, SYXK-2018-0019).

## Author Contributions

XX and JZ designed the experiments. LZ and MD performed the experiments. LZ analyzed the data and wrote the original draft. XX, JZ, QZ, and ML reviewed the manuscript. All of the authors had final approval of the submitted version.

### Conflict of Interest Statement

The authors declare that the research was conducted in the absence of any commercial or financial relationships that could be construed as a potential conflict of interest.
