# Supplementary material for: A Possible Mechanism: Genistein Improves Metabolism and Induces White Fat Browning Through Modulating Hypothalamic Expression of Ucn3, Depp, and Stc1
Source: Front Endocrinol (Lausanne). 2019 Jul 16;10:478. doi: 10.3389/fendo.2019.00478 (PMC6646519; doi:10.3389/fendo.2019.00478)
Supplement: Supplementary file 1 [file Data_Sheet_1.PDF]

Supplementary Material

Table S1. The nutritional compositions of three types of diet. CON, normal control diet; HF, high-fat diet; HFG, high-fat diet with genistein.

Ingredients	CON(g)	HF(g)	HFG(g)
Casein	200	258	258
L-Cystine	3	4	4
Corn Starch	397	0	0
Maltodextrin	132	162	162
Sucrose	100	89	89
Cellulose	50	65	65
Soybean Oil	0	0	0
Corn Oil	70	32	32
t-Butylhydroquinone	0.014	0	0
Mineral Mix S10026	0	13	13
MinarelMix	35	0	0
Vitamin Mix	0	13	13
Vitamin Mix	10	0	0
Choline Bitartrate	2.5	2.6	2.6
Lard	0	316.6	316.6
DiCalcium	0	16.8	16.8
Calcium Carbonate	0	7	7
Potassium Citrate, 1	0	21	21
FD&C Blue Dye #1	0	0.06	0.06
Genistein	0	0	0.25
Total	1000	1000	1000

Table S2. The primers sequences of the nine genes.

Genes	Forward	Reverse
Ucn3	AAGCCTCTCCCACAAGTTCTA	GAGGTGCGTTTGGTTGTCATC
DEPP	CCCCATTTGCCAACGATTCG	GCTGACAGATACACCTGACGTAG
Stc1	ACGAGGCGGAACAAAATGATT	TGCACTTTAAGCTCTCTTTGACA

Ucp1	TCTCTGCCAGGACAGTACCCAA	GAGTCGCAGAAAAGAAGCCACAA
Cidea	TGACATTCATGGGATTGCAGAC	GGCCAGTTGTGATGACTAAGAC
PGC1αα	TATGGAGTGACATAGAGTGTGCT	CCACTTCAATCCACCCAGAAAG
PPARα	AGAGCCCCATCTGTCCTCTC	ACTGGTAGTCTGCAAAACCAAA
PPARγ	TCGCTGATGCACTGCCTATG	GAGAGGTCCACAGAGCTGATT
β-actin	GGCTGTATTCCCCTCCATCG	CCAGTTGGTAACAATGCCATGT

Ucn3 (urocortin 3); *Depp* (decidual protein induced by progesterone); *Stc1* (stanniocalcin1); *Ucp1* (uncoupling protein 1); *Cidea* (cell death activator); *PGC1α* (peroxisome proliferator-activated receptor gamma coactivator 1-alpha); *PPARα* (peroxisome proliferator-activated receptor alpha); *PPARγ* (peroxisome proliferator-activated receptor gamma).
